# A consolidated review of commercial-scale high-value products from lignocellulosic biomass

**DOI:** 10.3389/fmicb.2022.933882

**Published:** 2022-08-23

**Authors:** Bo Zheng, Shengzhu Yu, Zhenya Chen, Yi-Xin Huo

**Affiliations:** ^1^Key Laboratory of Molecular Medicine and Biotherapy, School of Life Science, Beijing Institute of Technology, Beijing, China; ^2^Center for Infectious Disease Research, School of Medicine, Tsinghua University, Beijing, China

**Keywords:** lignocellulosic biomass, pretreatment, bioenergy, biorefinery, biofuels

## Abstract

For decades, lignocellulosic biomass has been introduced to the public as the most important raw material for the environmentally and economically sustainable production of high-valued bioproducts by microorganisms. However, due to the strong recalcitrant structure, the lignocellulosic materials have major limitations to obtain fermentable sugars for transformation into value-added products, e.g., bioethanol, biobutanol, biohydrogen, etc. In this review, we analyzed the recent trends in bioenergy production from pretreated lignocellulose, with special attention to the new strategies for overcoming pretreatment barriers. In addition, persistent challenges in developing for low-cost advanced processing technologies are also pointed out, illustrating new approaches to addressing the global energy crisis and climate change caused by the use of fossil fuels. The insights given in this study will enable a better understanding of current processes and facilitate further development on lignocellulosic bioenergy production.

## Graphical Abstract

Overall schematic diagram of lignocellulosic biomass conversion into value-added products. 
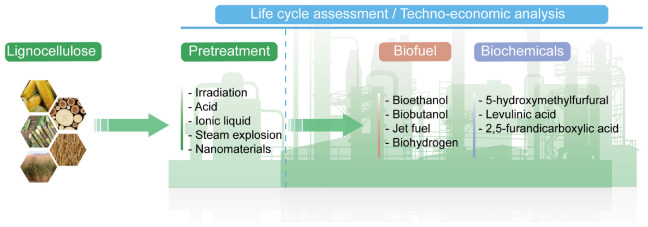

## Introduction

Global primary energy consumption has expanded rapidly in the last decades and reached its fastest rate of 2.9% annual growth since 2010. Oil and coal remain the most wildly used fuels and account for around half of the global energy consumption. To a very large extent, an above-average growth of fossil fuel consumption directly leads to the fastest expansion (2.0%) of carbon emissions, with a historic high of 33.1 gigatons of energy-related CO_2_ emissions in 2018 (BP, 2019; Usmani et al., 2020). The vast amount of released CO_2_ from fossil fuels resulted in a persistent temperature anomaly above the average temperature of the last 19th century, particularly in the prominent low-carbon scenarios for 2050 (Heleniak, 2021). Owing to the chasm between fossil fuel-reserved status and stringent climate targets, multiple scenarios have suggested that additional efforts are required to limit global CO_2_ emissions (Rogelj et al., 2018; WESP, 2020). According to the current oil price, the global crude oil consumption in 2018 was up to 4.6 billion tons, the highest level since 2010 (Dudley, 2018; Chai et al., 2021). Therefore, developing and promoting low-carbon energy sources are urgently needed to reduce the global carbon footprint and meet the net-zero-emission target.

Nowadays, plant biomass provides approximately 10% of global primary energy and is expected to mitigate the energy and environmental challenges (Ahmad et al., 2020). Among various kinds of plant biomass, lignocellulosic biomass is thought to have the greatest potential since it is widely available and potentially has a lower-cost per-unit energy than petroleum (Lynd et al., 2017; Ufodike et al., 2020). Lignocellulosic biomass, also termed as lignocellulose, is a carbon-neutral bioenergy feedstock and the most abundant bio-renewable material on the earth, which is considered the most abundant carbon-neutral resource that can reduce CO_2_ emission and solve the energy crisis. As an example, wheat straw is considered typical lignocellulose and mainly consists of cellulose, hemicellulose, and lignin. Currently, wheat straw is being produced in ever greater quantities, with the growth of global agriculture, with China annually contributing over 900 million tons of straw, mainly derived from corn, rice, and wheat (Han et al., 2020; Ma et al., 2020). However, most of wheat straw is openly burnt *in situ* for the preparation of the farmland and agriculture cycle, which will not only cause a tremendous waste of lignocellulosic energy, and also emits a large amount of greenhouse gasses (GHG) (Chai et al., 2021).

Based on the renewable property and huge economic benefits, lignocellulosic has been utilized in the synthesis of various high-value compounds. This review concentrated on the pretreatment technologies of lignocelluloses, including physical, chemical, physicochemical, biological, and other pretreatments, and discussed the bottlenecks and challenges of different pretreatment methods, with a special focus on new strategies to overcome pretreatment barriers. In addition, the generation of high-value-added products, especially biofuels, biohydrogen, and biomaterials from lignocellulosic raw materials is an attractive method to achieve the sustainable development. The current status and application prospects of bioethanol, biobutanol, 5-hydroxymethylfurfural (HMF), and levulinic acid (LA), which is produced from lignocellulosic biomass, are summarized and reviewed herein. Moreover, a comprehensive assessment of the environmental sustainability and economic feasibility risks of current strategies has been discussed with life-cycle assessment (LCA) and techno-economic analysis (TEA). We also proposed the research directions for further development of economically and ecologically viable methods for the production of biofuels and other value-added biological products from lignocellulosic biomass.

## Structural characteristics of lignocellulosic biomass

Some technological barriers still hinder the cost-effective pretreatment of lignocellulosic biomass, but robust research is going on to overcome those obstacles. Lignocellulosic biomass has evolved to protect plants from infertility and hydrolysis through the recalcitrant structure of cell walls. This robustness is mainly a result of the cross-linking between polysaccharides and lignin through complex linkages (Tarasov et al., 2018).

**FIGURE 1:**
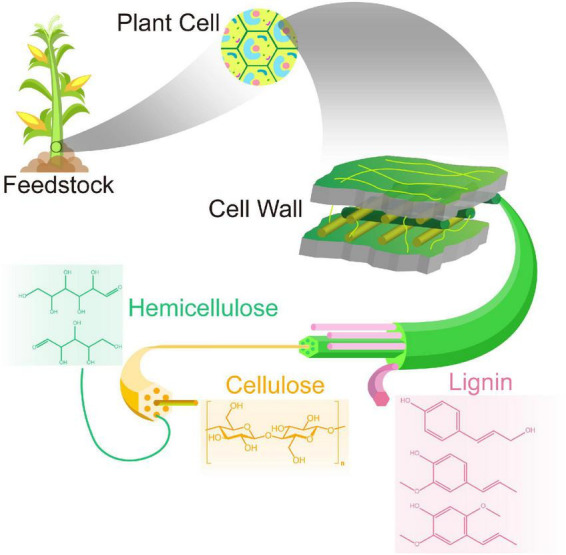
The fundamental structure and composition of lignocellulose biomass.

Lignocellulose is a complex matrix, consisting of polysaccharides, phenolic polymers, and proteins that, together, constitute the essential structure of the plant cell wall. In general, lignocellulosic materials from energy crops (e.g., corn) mainly consist of cellulose (35–50%), hemicelluloses (20–35%), and lignin (15–20%), as well as minor amounts of ash and extractive components (Zahoor, et al., 2021). Moreover, the lignin contents can reach 25% or higher in woody crops (e.g., willow) (Fonseca et al., 2020; Bonfiglio et al., 2021). However, these components are firmly intertwined and bonded together, forming a cross-linked three-dimensional polymer network of covalent or non-covalent bonds. Figure 1 shows the fundamental structure and composition of lignocellulosic biomass. Cellulose molecules are arranged in regular bundles or a random geometry, respectively, forming crystalline and amorphous regions. Cellulose and hemicellulose polymers in microfibrils are linked by hydrogen bonds and are protected by lignin. This stabilized structure confers lignocellulosic biomass with different degrees of resistance to various treatments, including enzymatic hydrolysis.

### Cellulose

Cellulose is linear polysaccharides, in which glucose molecules are linked together through β–(1→4) glycosidic bonds. The cellulose chains form a crystalline structure *via* hydrogen bonds and Van der Waals interactions, consequently aggregating into microfibrils and then fibers (Ashokkumar et al., 2022). This is the common structural feature of the primary cell wall of green plants and algae. Cellulose is the most abundant and available organic polymer in nature, but its proportion varies considerably with sources, for example, 90% in cotton fiber and 40–50% in wood. The high degree of crystallinity provides sufficient strength to cellulose but requires relatively rigorous condition (320°C and 25 MPa) to observe the crystalline-to-amorphous transformation in water, which hampers the subsequent saccharification for the biorefinery production.

### Hemicellulose

Hemicellulose is an amorphous and variable heteropolymer, containing approximately 500–3,000 sugar units of xylose, glucose, and galactose. It is cross-linked with either cellulose or lignin and further solidifies the cell wall (Figure 1). Of the hemicelluloses, the xylan (polymer of xylose) is predominated in hardwoods and cereal, whereas glucomannan is the major type occurring in softwoods. Although hemicellulose is widely available, the diversity of sugar units varies, depending on the source of lignocellulosic biomass, resulting in more challenging pretreatment compared to cellulose (Zoghlami and Paës, 2019). However, the differences in variability of the sugar unit, chain length, and structure of glycosyl side chains give hemicellulose a huge advantage over cellulose in aspect of for regioselective chemical and enzymatic modifications.

### Lignin

Lignin is the third major component of lignocellulosic biomass, bound to hemicellulose through covalent bonds, contributing to the rigidity and compactness of the plant cell wall (Kang et al., 2019). Lignin is made up of three types of phenylpropanoid monomers: 4-propenyl phenol, 4-propenyl-2-methoxyphenol, and 4-propenyl-2,5-dimethoxyl phenol (del Río et al., 2020).

Although lignocellulosic biomass has great potential economic value, pretreatment is an inevitable step for bioconversion to obtain monomeric sugars, which greatly increase the cost of bioprocessing (Sindhu et al., 2016; Sun et al., 2016b). The first step in a lignocellulosic facility is pretreatment for increasing the fractions of cellulose, hemicellulose, and lignin in the following hydrolysis step. It has been estimated that the pretreatment step accounts for 18–20% of the total project expense for the production of lignocellulosic ethanol, more than any other single step (Zhang et al., 2021). Furthermore, the associated cost of enzymes also prevents the production of bioenergy on an industrial scale (Saldarriaga-Hernández et al., 2020). These obstacles have led to the liquidation of many advanced biofuel startups, and the surviving companies have mainly focused on high-value-added products instead of fuels. Even though the global investment in biofuels and biochemicals prefers chemicals rather than fuels, the bioconversion of lignocellulose is confronted with both opportunities and challenges.

## Current technologies for the pretreatment of lignocellulosic

**FIGURE 2:**
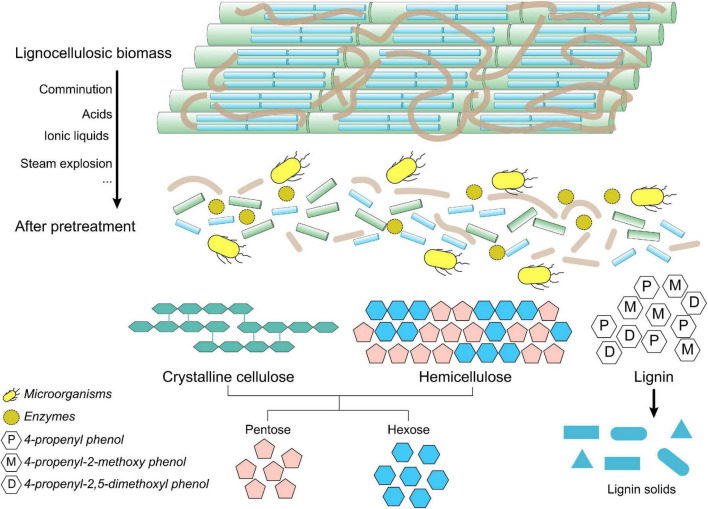
Changes in the structure of lignocellulose before and after pretreatment.

The industrial conversion of lignocellulosic biomass usually includes multiple steps, starting from the gathering and transportation of lignocellulose biomass, as well as the production of cellulase enzymes, followed by the pretreatment of lignocellulose biomass, hydrolysis of cellulose, fermentation of resulting monomer sugars, and, finally, treatment of effluent. Many pretreatment methods have been developed and optimized to maximally break down the cell walls, eliminate the hemicelluloses and/or lignin, increase the specific surface area, and so on, promoting the subsequent hydrolysis or depolymerization of cellulose. The main aim of the pretreatment is to deform the structure of lignin and reduce the crystallinity of cellulose to increase the contact area of the enzymes with cellulose during the process of hydrolysis. As shown in Figure 2, the recalcitrant structure of lignocellulose could be disrupted in the pretreatment process, resulting in easily hydrolyzed substrates for the subsequent enzymatic or microbial treatment.

The ideal pretreatment should be widely adaptable to different biomass sources, while also maintaining a low cost. The ideal pretreatment process should have (i) high-sugar yield and fermentation availability, (ii) minimal formation of toxic compounds and byproducts, as well as (iii) minimal energy requirements (Sindhu et al., 2016; Sun et al., 2016b; Bhutto et al., 2017). Several major pretreatment methods were compared in different ways and listed in Table 1.

**TABLE 1:** Comparison of different pretreatment methods on the lignocellulosic biomass.

Pretreatment method	Mode of action	Composition changed	Inhibitors generating	Sugar yield	Ethanol yield	Advantages	Disadvantages
Physical	Mechanical treatment	Decrystallization of cellulose	Low	89% (Sindhu et al., 2017)	−	Reducing the polymerization degree and crystallinity of the polymer	High energy consumption
	Irradiation	Decrystallization of cellulose	Low	99.7% (Liu et al., 2016)	70% (Liu et al., 2016)	Decreasing cellulose polymerization degree; reducing the use of chemicals	Expensive for the installation; high energy consumption
Chemical	Diluted acid	Removing hemicellulose (major); altering lignin structure (minor)	High	83% (Hsu et al., 2010)	−	Low cost; appropriate cellulose digestibility	High requirement for equipment
	Alkali	Removing lignin (major) and hemicellulose (minor)	Low	95% (Saratale and Oh, 2015)	46.5% (Saratale and Oh, 2015)	Low cost	High pollution and high chemical recovery cost
	Ionic liquids	Decrystallization of cellulose	High	75% (George et al., 2015)	73.4% (Yamada et al., 2017)	Strong ability in dissolving and decrystallization of the cellulose	High cost of ILs; difficult recovery
	Organosolv	Removing lignin (major) and hemicellulose (minor)	Low	45.1% (Li et al., 2013)	81% (Mesa et al., 2016)	Producing relatively pure lignin	High cost of organosolv; high quality lignin; solvent used maybe inhibitor for cell growth
	Oxidative	Removing lignin	Low			Delignification effectively	High cost of large amount of ozone
Physicochemical	Combined diluted acid/steam pretreatment	Removing hemicellulose (major); altering lignin structure (minor)	High	73–74% (Kuglarz et al., 2014)	55–58% (Kuglarz et al., 2014)	Low environmental impact	High requirement for equipment
	Liquid hot water	Removing hemicellulose	High	61.4% (Zhuang et al., 2016)	45.8% (Zhuang et al., 2016)	Low cost of reaction medium; less formation of inhibitors; appropriate cellulose digestibility	High energy and water requirement
	Ammonia fiber explosion	Removing lignin (major) and hemicellulose (minor); decrystallization cellulose	Low	76% (Zhuang et al., 2016)	58% (Zhuang et al., 2016)	No need for small particle size for efficacy	High cost of ammonia; high requirement for equipment
Biological		Removing lignin (major) and hemicellulose (minor)	Low	72.4% (Taha et al., 2015)		Low energy requirement; no chemical requirement; mild environmental conditions	Long process of biological pretreatment

Previous studies have developed different methods to pretreat lignocellulose with the aim to separate cellulose from lignocellulose and degrade lignocellulose to achieve a higher-sugar yield. The pretreatments should reduce the polymerization degree and crystallinity of cellulose, remove or degrade lignin and hemicellulose (or degraded lignin and hemicellulose), while also increasing the accessible surface area (Fei et al., 2020). Several selected methods are detailed below.

### Physical pretreatment

The aim of physical pretreatment is to enhance the accessible surface area by reducing the crystallinity and polymerization degree of cellulose, and thereby enhance the effectiveness of enzymatic hydrolysis (Chuetor et al., 2017, 2019). Physical pretreatment methods mainly included mechanical comminution (chipping, grinding, or milling) and irradiation (gamma rays, electron beam, microwave, etc.) (Karimi et al., 2013; Sun et al., 2016b; Bichot et al., 2020).

#### Mechanical comminution pretreatment

Mechanical comminution (MC) pretreatment alters the intrinsic internal structure of lignocelluloses and the extent of crystallinity by converting lignocellulosic biomass into particles of different sizes that are more amenable to enzymes. The pattern of the pretreatment defines the final particles size. For example, after the process of chipping, milling, and grinding, the particle size can reach 10–30 and 0.2–2 mm, respectively (Witaszek et al., 2020). Furthermore, the final particle size of lignocellulosic feedstocks is highly depended on the energy consumption of active mechanical comminution (Sun et al., 2016b). The microstructural and thermo-optical characteristics of lignocellulosic *Paulownia* biomass were altered by ultrafine grinding, providing significant benefits for subsequent hydrolysis and hydrogen production (Yi et al., 2020). Recently, it has been reported that combined biological and mechanical treatments could boost the bioethanol yield by up to 83%, resulting in an improvement of the overall substrate conversion of up to 131%. Accordingly, the combination of dry biological–mechanical pretreatments has great application potential in the future (Motte et al., 2015). At present, the high cost of single mechanical pretreatment impedes its large-scale application, but it can be combined with other pretreatment methods to great benefit.

#### Irradiation pretreatment

The cellulose in the lignocellulose biomass can be converted into microfibers, oligosaccharides with a low degree of polymerization, and even disaccharide *via* irradiation pretreatment. New irradiation pretreatment methods have been developed recently, including gamma irradiation, electron beam irradiation, microwave, and ultrasonic energy (Kumar et al., 2020). The gamma rays from radioactive decay of cobalt-60 or cesium-137 can penetrate biomass materials and decompose cellulose as well as lignin. A high conversion rate up to 72.68% for subsequent enzymatic hydrolysis could be achieved from agricultural straw after gamma irradiation at 800 kGy (Wu et al., 2020).

Ultrasound is sound waves with frequencies above 20 kHz, and has promising effect on cell wall disruption and accessible surface area changes. A recent study has suggested that ultrasound pretreatment led to significant improvement in lignocellulosic biomass solubility and biofuel yield (Marta et al., 2020). However, irradiation pretreatment, when used alone, has different defects, such as the formation of inhibitors and high energy consumption, and is now often combined with other pretreatment options. Several studies incorporating the acid and alkaline-mediated ultrasound pretreatment for increased biofuel yield have been reported. The application of ultrasound-assisted dilute acid pretreatment demonstrated that the combined method can eliminate the formation of inhibitors and increase the substrate utilization efficiency as well as bioethanol yield (Paramasivan et al., 2021). Similarly, ultrasound-assisted alkaline pretreatment was performed to achieve a high sugar content, resulting in a considerable yield of glucose after enzymatic saccharification (Ong et al., 2021). The combination of chemical or physical pretreatment with microwave was also employed to enhance yield with low doses of gamma irradiation. As found by Liu et al. (2021), alkali pretreatment combined with microwave irradiation of wheat straw could significantly increase the purity of cellulose up to 90.66%, and temperature is the most efficient factor among the process conditions.

### Chemical pretreatments

#### Acid pretreatment

The main objective of acid pretreatment is to decompose hemicellulose by disintegrating its bond with cellulose and partially degrade lignin, which can facilitate biomass fragmentation and production output (Mostofian et al., 2016). Both highly concentrated and dilute acids have been applied to preprocess a variety of lignocellulosic materials. However, pretreatments with concentrated acid require a large amount of acid, leading to corrosion of equipment, require additional recovery steps, and increase the cost (Kang et al., 2018). Even worse, low pH leads to unfavorable Pechmann reactions among lignin constituents, which leads to low lignin removal and can inhibit the subsequent enzymatic saccharification (Mostofian et al., 2016; Narron et al., 2016; Shuai et al., 2016). Therefore, diluted acid pretreatment is more widespread than the use of concentrated acid.

Pretreatment with diluted sulfuric acid with a concentration of 2–5% is usually carried out at a higher temperature in the range of 200–240°C, while a lower temperature is recommended for concentrated acid hydrolysis of biomass (Rezania et al., 2020). One study employed formic/acetic acid to pretreat beechwood at 104.2°C for 5 h and obtained 70.5% delignification. However, diluted acid pretreatment cannot remove the lignin, which was disadvantageous for subsequent steps (Martínez et al., 2015). These lignin droplets probably blocked the enzyme accessible to biomass or formed an ineffective combination with cellulose by intermolecular forces. A new bacteria-enhanced diluted acid pretreatment strategy has attempted to provide a solution to the above challenges. Using this approach, the enzymatic digestibility of rice straw was increased by 35–70 and 173–244% compared to diluted acid preprocessing and untreated biomass, respectively (Yan et al., 2017). This strategy was expected to be further developed to put into practical application. More attentions should be paid to reducing or eliminating inhibitors produced during the dilute acid pretreatment process.

#### Alkali pretreatment

Alkali pretreatment is the most frequently used method to get rid of lignin and hemicelluloses from lignocellulosic feedstocks and convert a large volume of lignocellulosic materials into lignocellulosic fibers. It was reported to have an advantage over acid pretreatment by causing less sugar loss and was shown to be more efficacious on agricultural residues than on wood biomass (Kim et al., 2016). The major aim of alkaline pretreatment is the disintegration of lignin, hemicellulose, and intermolecular ester bonds (Shi et al., 2021). Reagents, such as NaOH, Ca(OH)_2_(lime), Na_2_S, Na_2_CO_3_, NH_4_OH, and other alkaline reagents, have been employed to preprocess lignocellulosic feedstocks, whereby NaOH and Ca(OH)_2_ are the most commonly used (Sun et al., 2016b). Compared with liquid NaOH pretreatment, lime pretreatment is more cost-effective and environmentally friendly due to its easy separation with CO_2_ assistance. Moreover, lime pretreatment does not form inhibitors such as furfural and HMF (Mathew et al., 2011). The addition of an oxidant agent (O_2_/H_2_O_2_) in alkaline pretreatment [NaOH/Ca(OH)_2_] can optimize the results by increasing lignin elimination. Martínez et al. (2015) found that 90% (w/w) of glucan was converted into glucose by enzymatic action, and 50% of unwanted xylan was removed after pretreatment with NaOH. However, the main drawback of alkali pretreatment is the black liquor (wastewater containing unrecovered alkalis and dissolved lignin), as well as the high energy input for hydrolysis (Vu et al., 2020). To date, the combination of alkaline pretreatment with mechanical milling has been the most commonly used to improve the reactivity of lignocellulosic material and enhance the fermentable sugar yield (Schneider et al., 2017; Wang et al., 2018; Shen et al., 2020).

#### Ionic liquids pretreatment

Ionic liquid (ILs) is a kind of molten salt that is in liquid state at ambient temperature and consists of pairedions. ILs possess many advantaged characteristics, such as non-flammability, low vapor pressure, as well as good thermal and chemical stability (Bhutto et al., 2017). Since there is no toxic or flammable vapor generated, ILs are regarded as “green solvents” and have become rising stars in the field of lignocellulose pretreatment (Elgharbawy et al., 2016; Yoo et al., 2017; Das et al., 2021). ILs have a powerful ability to dissolve the inter- and intramolecular hydrogen bonds of lignocellulose, and their performance can be further improved by altering the negative ions as well as the alkyl residues of the positive ions (Xu et al., 2016). Some studies found that π-π bond can be formed between the imidazolium cation of the ILs and lignin, improving its degradation (Jiang et al., 2011; Shill et al., 2011). Furthermore, pretreating lignocellulose with certain ILs makes the biomass more accessible to enzymes due to structural and chemical alterations (Torr et al., 2016). A method using 1-ethyl-3-methylimidazolium acetate allowed the separation of lignocellulosic biomass into high-purity carbohydrate and lignin fractions, with an efficient ILs recovery (Chinwatpaiboon et al., 2021). In general, solvent recovery is usually complicated and requires high energy consumption, which limits the large-scale utilization of ILs. Further studies are, therefore, needed to optimize the process flow to drive economically feasible ILs methodologies for the pretreatment of lignocellulose.

#### Organosolv pretreatment

Organosolv (OS) fractionation was thought to be superior to other lignocellulose pretreatments methods as it efficiently generates pure products and uses an easily recovered solvent (Zhang et al., 2016). This process yields three independent components: water-free lignin, hydrous hemicellulose vapor, and a comparatively pure cellulose fraction (Zhao et al., 2017). Generally, various catalysts, such as formic, oxalic, acetylsalicylic, and salicylic acid, are added to enhance the performance of delignification and dissolution of the hemicellulose fractions (Li et al., 2016a; Zhang et al., 2016; Singh et al., 2022).

Ethanol is the most favored solvent for organosolv pretreatment due to its advantages, such as low cost, low boiling points, and easy recovery. However, for most low-boiling-point solvents, the pretreatment process is carried out at high pressure, which increases the operational costs (Bhutto et al., 2017). Conversely, employing violate organic solvents required exceedingly tight control due to intrinsic combustibility and explosion hazards (Bhutto et al., 2017). Previous studies have elucidated that the generation of undesired furfural and HMF can be reduced under mild pretreatment conditions at low temperature and pressure as well as neutral pH (Kim and Pan, 2010). Sun et al. (2016a) evaluated the commercial and industrial application of atmospheric glycerol organosolv pretreatment (AGO), and the results revealed good constituent selectivity, with the removal of approximately 70% lignin and hemicellulose, while preserving 94% of the overall cellulose. Based on the previous work, further research should focus on the optimization of pretreatment conditions for the final purpose of commercialization.

#### Oxidative pretreatment

Oxidative pretreatment is usually employed to bleach paper pulp (Sun et al., 2016b). In this process, nearly all lignin can be separated from lignocellulosic biomass without significant loss of cellulose and hemicelluloses, which optimizes the yield of fermentable sugars (Coca et al., 2016). Commonly used oxidation reagents include O_3_, O_2_, H_2_O_2_, ClO_2_, NaClO, and Cl_2_. The pretreatment is usually carried out at ambient temperature and pressure, thus avoiding the formation of toxic byproducts (Alvira et al., 2010). It was reported that the use of wet disk milling (WDM), followed by ozonolysis, was preferable for pretreating sugarcane bagasse and straw, resulting in a higher saccharification yield than WDM after ozonolysis (de Barros et al., 2013). Remarkably, a two-stage pretreatment enabled sugar recovery from corn stover with a low-enzyme loading (Li et al., 2016b). The pretreatment with diluted hydrochloric acid (0.7% wt hydrochloric acid at 120°C for 40 min), followed by Fenton oxidation (at room temperature for 12 h), resulted in the recovery of 81.0% xylose and 32.9% lignin. Ultimately, high sugar recovery was achieved with low-enzyme loading, but the large consumption of oxidation reagents made the process economically unviable (Travaini et al., 2016).

### Physicochemical pretreatments

#### Steam explosion pretreatment

Steam explosion is the most commonly used thermo-mechanic-chemical preprocessing method for lignocellulosic feedstocks, which allows the opening of the lignocellulosic structure *via* the combination of mechanical forces and chemical function (Verardi et al., 2018). The most important factors that determine the results of steam explosion are section size, temperature, dwell time, and the joint effect of both temperature and time (Sarker et al., 2021). López-Linares et al. (2015) showed that the optimal conditions for the maximization of enzymatic hydrolysis yield were 215°C and 7.5 min, leading to a maximum glucose yield of 72.3% from rapeseed straw. Wang et al. found that autohydrolysis mainly occurred on the hemicelluloses and the amorphous region of cellulose at a shorter steam pretreatment time. Prolonged incubation time could benefit the depolymerization of the crystalline region of cellulose (Smichi et al., 2020). Due to large amount of fermentation inhibitors generated under the condition of violent steam explosion, various cleaners, such as SO_2_ and H_2_SO_4_, have been applied to enhance the separation of hemicelluloses under relatively mild conditions (Sun et al., 2016b).

Steam explosion pretreatment has several favorable characteristics, such as high energy efficiency, low environmental impacts, being suitable for large particle size, and requiring no external chemicals (Bhutto et al., 2017). Furthermore, high sugar recovery can be achieved because the material is quickly heated by steam so that only small amounts of fluid remain in the equipment. However, the numerous inhibitors from sugar and lignin degradation, such as furfural and phenolic compounds, are the main disadvantage of steam explosion pretreatment. These compounds can be removed by washing the pretreated biomass with large amounts of water but raising the issue of loss of sugar and reprecipitation of lignin. Nonetheless, steam explosion pretreatment has been proved to be an effective pretreatment process for the production of various bioproducts. A remaining challenge of this process is how to minimize the production of inhibitors.

#### Liquid hot water pretreatment

Liquid hot water (LHW) pretreatment is also a hydrothermal treatment method related to the steam explosion pretreatment, albeit using liquid hot water rather than steam to preprocess lignocellulose. As a consequence of high-level decomposition of cellulose when the temperature exceeds 240°C, the hydrothermal temperature is usually fluctuated in the range of 160–240°C (Cao et al., 2014; Sun et al., 2014). The primary purpose of the liquid hot water is to degrade the hemicellulose and make it more accessible to enzymes. To minimize the produce of inhibitors, the pH should be maintained between 4 and 7 during the pretreatment, as hemicellulosic sugar is mostly maintained at a low degree of polymerization under these conditions (Chen et al., 2014b). In recent studies, several catalytic agents have been tentatively applied to assist the separation of hemicelluloses or lignin, such as dilute alkali and H_2_SO_4_ (Yao et al., 2018; Yang et al., 2019; Ladeira Ázar et al., 2020).

Overall, liquid hot water pretreatments had the advantages of high productivity, no catalysts requirement, low-cost installation, relatively high hemicellulose recovery, and low formation of inhibitors. However, high water and energy demand due to the high temperature and pressure as well as the relatively limited output of fermentable sugars are remaining challenges, which required further exploration.

#### Ammonia fiber explosion pretreatment

Ammonia fiber explosion (AFEX) exposes lignocellulosic materials to liquid ammonia at 60–100°C and 250–300 psi for an unequal length of time, followed by a rapid pressure release (Zhao et al., 2020). This process can evidently enhance enzymatic hydrolysis because liquid ammonia causes the expansion of lignocellulose and alters the crystal structure of cellulose, while the rapid release of pressure ultimately results in the physical disintegration and crystallinity attenuated of lignocellulosic biomass (Sun et al., 2016b). However, this process cannot remove significant amounts of lignin and hemicellulose from lignocellulose. Accordingly, this method allows low lignin preservation, reducing the inhibition of enzymes and promoting enzyme accessibility, dramatically reducing enzyme loadings. AFEX was reported to produce 36-fold-less furans and 100-fold-less carboxylic acids than dilute acid pretreatment, allowing the direct delivery to fermentation without any detoxification (Chundawat et al., 2010). AFEX has achieved high conversion rates (80–97%) of various biomass sources, such as corn stover, wheat straw, and rice straw (Momayez et al., 2018; Qiao et al., 2018; Wang et al., 2019).

Ammonia fiber explosion is a forward-looking pretreatment process with several virtues, such as being harmless to the environment, having low energy consumption, mild-reaction conditions, and negligible production of inhibitors. Furthermore, most of the ammonia can be recycled back into the process reactor to reduce the operating input. Nevertheless, the expensive liquid ammonia is less competitive for industrial applications than some of the other commonly used solvents.

#### Nanobiotechnology pretreatment

Considering disadvantages of conventional lignocellulosic biomass pretreatment methods, such as the cumbersome and costly treatment process, serious environmental pollution, and the limitations in reducing the polymerization rate and crystallinity of cellulose (Chandel et al., 2022), it is crucial to develop economical and efficient pretreatment methods for lignocellulose. Recently, utilizing of nanobiotechnology for lignocellulosic biomass pretreatment has gained widespread attentions, which is considered to be an effective solution to overcome the bottlenecks. Nanomaterials with magnetic properties could be recovered from the reaction mixture for reuse by increasing the magnetic field, indicating a cost-effective and ideal pretreatment method (Sajid and Płotka-Wasylka, 2020). Due to the properties such as the high-specific surface area, thermal stability, and high specificity, nanomaterials have been utilized for the immobilization of enzymes, which could enhance their reusability and the production of immobilized proteins. Nanotechnology is mainly applied to the pretreatment of lignocellulosic biomass by acid-functionalized magnetic nanoparticles mediated and nano-shear hybrid alkaline (NSHA). It has been reported that the pretreatment of 500 mg g^–1^ of sugarcane bagasse with two types of acid-functionalized magnetic nanoparticles, alkyl sulfonic acid, and butyl hydroxy acid yielded 18.83 and 18.67 g L^–1^ sugar, respectively (Mahajan et al., 2020). Deng et al. utilized the NSHA method for pretreatment of corn stover biomass to improve biofuel production. Approximately, 70% of cellulose was converted, which significantly facilitates efficient biofuel production (Deng et al., 2018).

With rapid development of the global industry, fossil energy is becoming increasingly scarce. The acquisition of bioenergy from renewable resources plays a significant part in the eco-friendly society, which promotes green and sustainable development. At present, various nanomaterials, such as magnetic nanoparticles, zinc oxide nanoparticles, and magnesium oxide nanocatalysts, have been utilized in improving the production of renewable bioenergy from lignocellulosic biomass (Dey et al., 2022; Govarthanan et al., 2022).

In conclusion, the application of nanotechnology could effectively improve the efficiency of lignocellulosic pretreatment and reduce the environmental pollution. The utilization of magnetic nanoparticles for lignocellulosic biomass pretreatment or immobilization of enzymes is considered as a cost-effective solution. Meanwhile, nanobiotechnology shows great potential for efficient production of biofuels and other high-value-added compounds.

### Biological pretreatment

Biological pretreatment is an environmentally friendly technique, and has attracted increasing interest for promoting the enzymatic digestion of lignocellulosic materials in ethanol production processes (Batog and Wawro, 2021). Commonly employed microorganisms, such as brown, white, and soft rot fungi, perform their function on depolymerizing lignin and hemicelluloses in lignocellulosic biomass (Saritha and Arora, 2012; Sindhu et al., 2016). Brown-rot fungi mainly digest cellulose, while the white- and soft-rot fungi utilize both cellulose and lignin (Meehnian et al., 2017; Couturier et al., 2018). Notably, white-rot fungi are the most popular choice for the biological pretreatment of lignocellulosic biomass (López-Abelairas et al., 2013; Machado and Ferraz, 2017). A comprehensive review has discussed various facets of biological pretreatment, related enzymes, factors influencing biological pretreatment, as well as future perspectives (Singh, 2021).

Although biological pretreatment offers many advantages, such as low-capital investment, using less energy, requiring no externally added chemicals, having no inhibitor production, and mild pretreatment conditions, it is hindered by the very long incubation time (several weeks) and the relatively low hydrolysis yields. Screening or engineering of more powerful microorganisms, as well as optimizing the fermentation methods, is certainly a worthwhile research direction for the future.

### Other pretreatments

Novel and updated pretreatment methods for lignocellulose include CO_2_ explosion pretreatment, SO_2_ explosion pretreatment, N_2_ explosion pretreatment, supercritical fluid pretreatment, and wet oxidation and hydrogen peroxide-acetic acid pretreatment (Wi et al., 2015; Lee et al., 2020; Arumugham et al., 2022). However, ongoing efforts to apply these methods have not yet resulted in economically viable industrial processes.

A recent study has assessed the techno-economics of four different pretreatment processes, including steam explosion, dilute sulfuric acid, AFEX and biological pretreatments. The authors suggested that steam explosion and sulfuric acid pretreatment methods might be good alternatives at the present state of technology, but further research is needed to make them industrially competitive (Baral and Shah, 2017). Similarly, scenarios for dilute acid, liquid hot water, and AFEX methods were studied, and the results suggested the best scenarios, which achieved total annual costs reduction of 19.6%, as well as Minimum Bioethanol Selling Prices reduction of 30.2% (da Silva et al., 2016). Specifically, liquid hot water pretreatment (SCN-L3) achieved the best minimum bioethanol selling prices, with savings of 30%. However, these results should be used as guidelines and are not suitable as standards because the experimental designs and reporting results did not provide adequate consistency to allow comparison and identification of the best protocols.

## High-value products from lignocellulosic biomass

**FIGURE 3:**
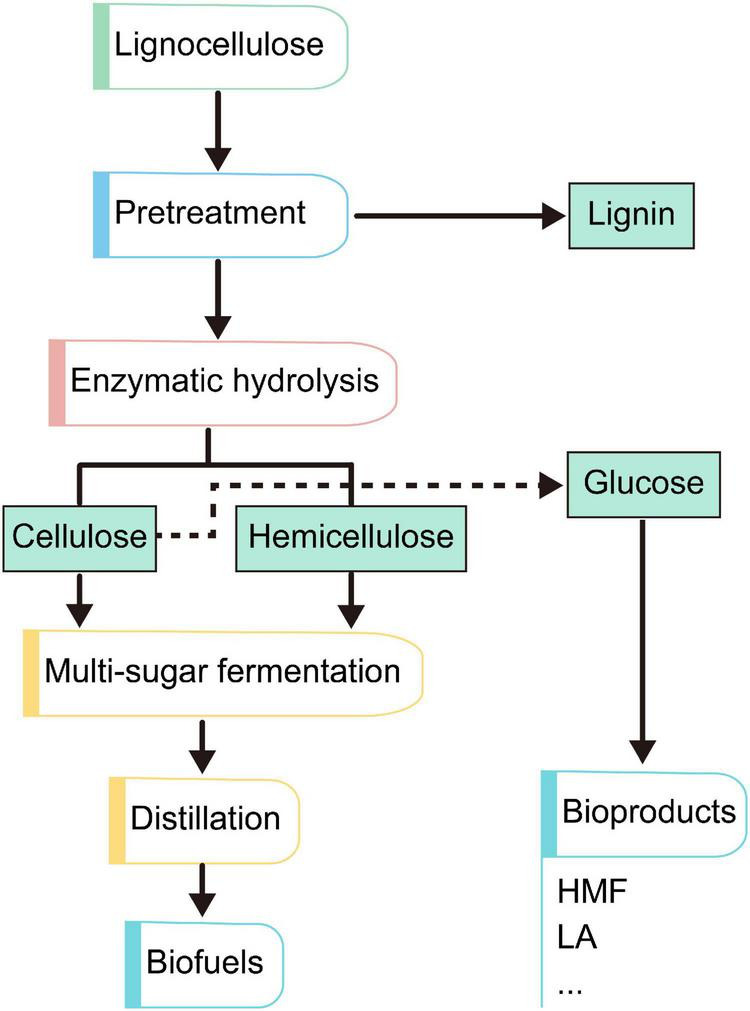
General routes of formation of different value-added products from lignocellulosic biomass.

In recent years, lignocellulosic has attracted increasing attentions biomass as a renewable and sustainable energy. The emerging technologies based upon the concept of biomanufacturing contribute a wide range of bioproducts, including biofuels, biohydrogen, biomaterials, and a series of biochemicals from fermentation and refining steps (Figure 3). Therefore, lignocellulosic biomass provides an attractive alternative for the production of biofuels and chemicals in a sustainable and eco-friendly society, which had led to its reorganization as a valuable commodity all over the world. Table 2 shows some value-added products that have been successfully produced from the different components of lignocellulosic biomass.

**TABLE 2:** Lignocellulosic derivatives production with different pretreatment methods.

Product	Biomass	Pretreatment	Conditions	Microorganism	Yield	Reference
Ethanol	Wheat straw	Ionic liquid	[TEA][HSO_4_]; biomass/solvent ratio of 1:5 g/g; 130°C; 0.5–3 h	*Saccharomyces cerevisiae* (PTCC 5052)	46.0 g/100 g wheat straw	Ziaei-Rad et al., 2021
Butanol	Sugarcane bagasse	Acid	1.5% H_2_SO_4_; solid/liquid ratio of 1:10; 160.47°C; 5 min	*Clostridium acetobutylicum* ATCC824	16.51 g L^–1^	Mondal et al., 2022
Biodiesel	Corncob	Alkali	3% NaOH; solid/liquid ratio of 1:8; 121°C; 30 min	*Trichosporon oleaginosus* DSM 11815	26.74 g L^–1^	Grubišić et al., 2021
2,3-Butanediol	Sugarcane bagasse	Acid	1% H_2_SO_4_ ; solid/liquid ratio of 1:6; 121°C; 30 min	*Enterobacter aerogenes* EMY-22_M1Gb	128.4 g L^–1^	Kim et al., 2020
1,3-propanediol	Cactus cladode	Acid	1.5% H_2_SO_4_ ; solid/liquid ratio of 1:3; 121°C; 1 h	*Lactobacillus diolivorans* DSM 14421	0.75 g g^–1^	de Santana et al., 2021
Xylitol	Corncob	Acid	0.5% H_2_SO_4_ and 1.5% (w/w) H_3_PO_4_; solid/liquid ratio of 1:3; 128°C; 1h	*Kluyveromyces marxianus* CICC 1727-5	24.2 g L^–1^	Du et al., 2020
Methane	Potato crop residues	Organosolv	Solid/liquid ratio of 1:10; 180°C; 1 h	−	196.0 mL g^–1^	Soltaninejad et al., 2022
Hydrogen	Corn stover	Mechanical comminution	10 mm zirconia balls; ball/volume ratio of 2:1; 380 rpm; 6 h	Photosynthetic mixed consortium HAU-M1	425 mL	Tahir et al., 2021
Itaconic acid	Bamboo	Alkali and steam explosion	Alkali: 2% NaOH; solid/liquid ratio of 1:12; 25°C; 48 h Steam explosion: 1.0 MPa for 6 min	*Aspergillus terreus* AtYSZ-38	41.54 g L^–1^	Yang et al., 2020
Succinic acid	Sugarcane bagasse	Alkali	2% NaOH; solid/liquid ratio of 1:20; 80°C; 2 h	*Saccharomyces cerevisiae Actinobacillus succinogenes*	22.1 g L^–1^	Xu et al., 2021
Lactic acid	Beechwood	Organosolv	OxiOrganosolv; solid/liquid ratio of 1:10; 2 h	*Lactobacillus delbrueckii*	62 g L^–1^	Karnaouri et al., 2020
Butyric acid	Spent coffee grounds	Acid	0.04 M H_2_SO_4_; solid/liquid ratio of 1:10; 121°C; 40 min	*Clostridium tyrobutyricum* ATCC 25755/ketp	34.3 g L^–1^	Yang et al., 2020
Propionic acid	Sweet sorghum bagasse	Acid	Concentrated H_2_PO_4_; 130 g L^–1^ biomass concentration; 50°C; 43 min; pH 5.0	*Propionibacterium freudenreichii* DSM 4902	0.45 g/g sugar	Ammar et al., 2020
Gluconic acid	Corn stover	Acid	2.5% H_2_SO_4_; solid/liquid of 2:1 (w/w); 175°C; 5 min	*Gluconobacter oxydans* DSM 2003	0.761 g g^–1^ sugar	Zhou et al., 2019

Bioethanol is a well-known biofuel used in the transportation sector worldwide by far, which can be produced by fermentation of a variety of feedstocks. Studies focusing on the bioconversion of lignocellulose to bioethanol provide different strategies for improving the yield, aiming to pave the way for industrial-scale bioethanol production. Compared with bioethanol, biobutanol has a higher-energy content and a higher-octane number, resulting in a drop-in fuel alternative, which can be used alone or blend with gasoline in any concentration. Another important liquid biofuel is biodiesel, a form of diesel fuel generated by a chemical transesterifying process that combines vegetable oils, animal fats, and alcohol in the presence of a catalyst. To date, biodiesel has been studied extensively and has been produced on a commercial scale through numerous advancements in pretreatment, catalysts, and reactor optimization (Zhang et al., 2019; Mohiddin et al., 2021).

These liquid biofuels emit GHG upon combustion and, therefore, are not considered as preferable energy sources yet. Biohydrogen, as a fuel, has been seen as one of the cleanest energy carriers as it is renewable, carbon free, and has no emission of GHGs from combustion (Abdin et al., 2020). Usually, conversion of lignocellulosic biomass to hydrogen can be accomplished by thermochemical methods (gasification or pyrolysis) and the biological process (bio-photolysis, photo fermentation, or dark fermentation) (Singh et al., 2021). Apart from these routes, nanotechnology has begun to gain interest for promoting the biohydrogen production. Many studies have been reported to investigate the effect of different nanoparticles on biohydrogen production, and have revealed that nanoparticles help the cell to transfer electron faster during redox reaction (Tahir et al., 2021).

Although bioethanol is the main product in acetone-butanol-ethanol (ABE) fermentation by Clostridium species, other products, such as 2,3-butanediol, xylitol, lactic acid, and butyric acid, can be obtained by metabolic engineering and adaptive evolution of candidate strains (Araújo et al., 2021; Lee et al., 2021; Zou et al., 2021; Fu et al., 2022).

In addition, different enzymes used during the hydrolysis process of lignocellulosic biomass could be obtained from various microbes (Table 3). For example, Lignicell Refining Biotechnologies Ltd. (formally Laihe Rockley Bio-chemicals Co. Ltd., a sino-foreign joint venture company) reported an annual production of 40,000 tons of ABE in 2012 (Jiang et al., 2015). Moreover, compared with the fossilized one, the price of n-butanol ($1.32 kg^–1^) was very competitive compared to the price of the petrochemical alternative ($1.52 kg^–1^).

**TABLE 3:** Enzymes for lignocellulose biomass hydrolysis.

Enzyme	Substrate	Source	Optimal pH/Optimal temperature (°C)	Novelty/Scope	Reference
PersiCelXyn1	Cellulose, xylan	Rumen microbiota	5.0/50	Bifunctional enzyme High efficiency in degrading various biomass substrates	Ariaeenejad et al., 2021
Swollenin-xylanase	Cellulose	*Trichoderma reesei*	5.5/55	Artificial fusion enzyme with increased hydrolysis capacity	Du et al., 2021
PersiCel1/2	Cellulose	Camel rumen microbiota	8.0/60 for PersiCel1, 5.0/50 for PersiCel2	A novel thermostable cellulase cocktail	Maleki et al., 2020
CMCase	Cellulose	*Bacillus amyloliquefaciens* SS35	4.0/65	Stable in the acidic range Economical for SSF	Singh et al., 2020
Endoglucanase	Cellulose	*Spirochaeta thermophila*	5.0/70	High thermostability little activity with low molecular weight substrates	Hämäläinen et al., 2021
α-Amylase	Polysaccharides	*Geobacillus* sp. GS33	6.0/70	High thermostability Neutral pH optimum	Burhanoğlu et al., 2020
Laccase	Lignin	*Botrytis cinerea* 241	5.5/60	High thermostability High salt tolerance Broad substrate specificity	Radveikienė et al., 2021
Versatile Peroxidase	Lignocellulose	*Citrus sinensis*	2.4/18	Higher substrate affinity to manganese sulfate	Rai et al., 2020
Manganese peroxidase	Lignin	*Ganoderma lucidum*	−	Degrade a wide range of phenolic dyes	Xu et al., 2017
Aryl-alcohol oxidase	Lignin	Moesziomyces antarcticus	6.0/57.5	Mild reaction conditions	Lappe et al., 2021
Lignin peroxidase	Lignin	*Pleurotus pulmonarius*	3.0/30	High specific activity	Giap et al., 2022

Novel value-added products, such as HMF, LA, and 2,5-furandicarboxylic acid (FDCA), have recently been produced from lignocellulosic biomass *via* advanced reaction systems and selective catalytic processes. HMF is produced industrially on a modest scale, and is mainly used as a flavoring agent in the food industries. Important derivatives, such as furfuryl alcohol and 2-methyltetrahy-drofuran, have great potential in the synthesis of furan resins. It was reported that lignocellulose can be directly converted into HMF with an approximate 49% yield in a seawater-tetrahydrofuran biphasic medium (Guo et al., 2018). As a keto acid generated mainly from HMF, LA has high reactivity and wide functionality due to the presence of ketone and carboxylic functional groups, and was recognized as an important precursor for the synthesis of biofuels. In 2015, GF Biochemicals began the large-scale commercial synthesis of LA based on the acid catalysis process. The commercial production of FDCA was first initiated at a pilot plant scale in 2011 by Avantium, using catalytic oxidation processes with a yield of approximately 50%.

However, most of startup companies have been shut down because of financial issues caused by the price depreciation of n-butanol. Those that have survived still struggled with various problems, such as less cost-effective pretreatment, troublesome inhibitors, as well as low-yield productions. In this section, we have summarized the recent advancement and challenges of converting lignocellulose to high-value products, including bioethanol, biobutanol, HMF, and LA.

### Bioethanol

Ethanol (C_2_H_5_OH) is the most abundant biofuel and is being considered as a potential substitute in petroleum-derived transportation industries. First-generation commercial ethanol production was based on starch and sugar-derived feedstocks, which resulted in the food-versus-fuel debate over bioethanol and a consequent food price crisis. The conversion of lignocellulosic biomass into bioethanol is of great interest and is considered the second generation of bioethanol due to its low environmental impacts and wide available resources that do not compete with food production. Generally, the production of bioethanol from lignocellulosic biomass can be achieved *via* biochemical and thermochemical conversion. Both routes result in simple oligomers of lignin, hemicellulose, and cellulose *via* degradation, but the subsequent conversion technologies are not similar. The Fischer-Tropsch process is a heterogeneous catalytic reaction that can gasify biomass to produce syngas (CO and H_2_), which can be chemically converted into ethanol, butanol, and green diesel (Snehesh et al., 2017). An enzymatic hydrolysis process was integrated with the fermentation of reducing sugars in the same device, termed as simultaneous saccharification and fermentation (SSF), resulting in the biological transformation of sugars (produced in the procedure of the enzymatic hydrolysis of cellulose) into ethanol. Therefore, the final product inhibition of cellulase caused by the accumulation of sugars was neutralized due to the presence of yeast and the cellulolytic enzyme complex. In addition, the entire process demanded a single fermenter, thereby economizing the investment costs. However, there was one highlighted problem that the optimal conditions of saccharification and fermentation were not compatible. Research on superior reactor installation allowed the fermentation to the ethanol process based on the technology of fed-batch seemingly to be a prospective means to enhance the end output of ethanol. To this end, various studies optimized the simultaneous saccharification and fermentation process.

Paschos et al. proposed a semi-consolidated process involving two microorganisms (*Saccharomyces cerevisiae* and *Fusarium oxysporum*) for the fermentation of a highly viscous liquefied material *via* an in-house free-fall mixing reactor. The authors claimed that the addition of solid *Fusarium oxysporum* culture resulted in an ultimate ethanol concentration of 58 g L^–1^, an increase of up to 19% compared to the control (Paschos et al., 2015). Choudhary et al. (2017) isolated ten yeast strains from diverse sources and evaluated their production potentials, among which *S. cerevisiae* JRC6 showed the best fermentation performance, providing a promising alternative for simultaneous saccharification and fermentation processes. However, the pre-hydrolysis time was not systematically assessed in most studies, which is an important step before SSF and impacts on yield, productivity as well as ethanol concentration. A recent study has evaluated a pre-hydrolysis and SSF performance and maximized the ethanol concentration *via* multi-criteria optimization, resulting in 290 L of ethanol per ton of pretreated sugarcane straw (Pratto et al., 2020). In addition, a suitable microbial strain that is capable of converting lignocelluloses efficiently has been requested in the bioethanol production industry. The novel genetic manipulation strategies allow overexpression and editing of genes in different microbial candidates to gain enhancement of ethanol yield, productivity, and tolerance. *Escherichia coli* is one the most common model bacteria and has been engineered for the selective production of bioethanol *via* genetic manipulation. In recent years, different studies have been performed to optimize the substrate utilization, ethanol tolerance, and robustness of engineered strains through several different aspects. Recently, a metabolic engineered *E. coli* strain WDH-LF has been given the positive impact on ethanol production by the deletion of *frdD* and *ldhA*. Compared to the parental strain, the resultant strain redirected carbon flux to ethanol production and increased up to 167% higher-ethanol production from glucose (Lopez-Hidalgo et al., 2021). Moreover, other promising microorganisms, including *Cyanobacteria*, thermophilic bacteria, and *Zymomonas mobilis*, also have employed to improve ethanol production (Rahayu et al., 2020; Sarkar et al., 2020; Roussou et al., 2021).

### Biobutanol

Butanol (C_4_H_9_OH) is an important chemical precursor in many industries, as well as a potential direct substitute for petrochemical gasoline. Butanol is a highly flammable straight-chain alcohol with a boiling potion of 117°C. Although biobutanol and bioethanol can be produced *via* similar fermentation of lignocellulosic biomass, biobutanol is a superior biofuel compared to bioethanol due to its good miscibility with gasoline, non-hygroscopic nature, low-Reid vapor pressures, lower volatility than other traditional fuels, fewer emission, and higher-energy content than ethanol (Pugazhendhi et al., 2019). The leading biobutanol producers, such as Butamax and Gevo, have been devoting significant research efforts to the replacement of fossil fuels (Wang et al., 2017).

The most common microorganisms for biobutanol synthesis are anaerobic spore-forming bacteria, including *Clostridium acetobutylicum*, *Clostridium beijerinckii*, and *Clostridium saccharobutylicum*, *Clostridium saccharoperbutylacetonicum* (Das and Maiti, 2022). Generally, solventogenic Clostridia can synthesize butanol *via* the ABE fermentation process from renewable lignocellulosic feedstocks, but this route has been characterized byproduct inhibition. A recent study has revealed that overexpression of the efflux pump gene *srpB* boosted robustness of *C. saccharoperbutylacetonicum*, resulting in the growth capability in the high concentration of butanol and inhibitors (e.g., furfural and ferulic acid) (Jiménez-Bonilla et al., 2020). The biobutanol production *via* the ABE route faces high-economic risk due to the overproduction of acetone, which is lower boiling points and corrosive byproducts (Cai et al., 2022). This unfavored compound can be substantially converted into isopropanol through the isopropanol-butanol-ethanol pathway in *C. beijerinckii* strain, or be eliminated in engineered acetone-free producers, such as *S. cerevisiae* and *E. coli* (Ferreira dos Santos Vieira et al., 2020; Nawab et al., 2020; Veza et al., 2021).

However, the key challenges affecting the large-scale synthesis of biobutanol are mainly attributed to pretreatment costs, low yield, and downstream recovery costs. To bridge the gap between current production efficiency and that required for commercial viability of biobutanol, further research efforts should be focused on improving the production strains using novel numerous molecular tools.

The evolution of bioinformatics, high-throughput methods, and CRISPR/Cas9 system could overcome the barriers to pretreatment processing and improve the generation of bioproducts from lignocellulose biomass. Luo et al. (2020) elucidated the physiological mechanism of *Clostridium acetobutylicum* ATCC824 in response to phenolic inhibitors, which generated in hydrolysates during the lignocellulosic biomass pretreatment process by the combination of kinetic and transcriptional analysis, and explored the influence of phenolic stress on the production of butanol. Meanwhile, the transcriptional analysis revealed that the genes expression profile related to membrane transporters has been affected by phenolics, which has important guiding significance for achieving efficient synthesis of biobutanol from lignocellulose. Furthermore, genetic engineering of host strain proves to be an important research direction to produce high-value products from lignocellulosic biomass. Recently, different target genes have been modified in several species to improve the efficiency of saccharification and enhance the recalcitrance (Va et al., 2021). The butyrate fermentative supernatant has been utilized to produce butanol in *Clostridium acetobutylicum/Saccharomyces cerevisiae* co-culture system, and the increment reached 46.8% compared to control (Luo et al., 2017). Zhang et al. (2018) developed an inducible endogenous CRISPR-Cas9 platform, which generated mutant Δcat1::adhE2, revealing great potential of economic butanol production.

### 5-Hydroxymethylfurfural

5-Hydroxymethylfurfural (C_6_H_6_O_3_) has been investigated as a versatile biobased C5 and C6 platform molecule for the biorefinery, but high-production costs have hindered its development on an industrial scale (Delbecq et al., 2018). HMF is one of the most important C6-platform molecules for the production of numerous industrial chemicals, including LA, furfuryl alcohol, and furandicarboxylic acid (Li et al., 2019; Xu et al., 2020). HMP is produced industrially *via* the degradation of hexoses or through acid-catalyzed dehydration of cellulose (Li et al., 2019; Portillo Perez et al., 2019). The primary reason for the low yield of HMF is the rapid conversion to LA in aqueous solvents. Accordingly, recent studies have attempted to promote the synthesis of HMF and related products by employing homogenous and heterogeneous catalysts. Many studies investigating the heterogeneous acid-catalyzed conversion of fructose into HMF suggested that the high selectivity to HMF is controlled by the acid type, fructose tautomer distribution, and the solvent type (Svenningsen et al., 2018). A high-HMF yield from glucose approaching 60% was achieved by regulating the ratio of Lewis-to-Brønsted acid sites, with an optimal ratio of 0.3 (Swift et al., 2016). A higher selectivity of HMF (90%) synthesis from fructose, reaching 90%, was attained by using a sulfonic acid-functionalized acidic catalyst in dimethyl sulfoxide solvent at 120°C (Chen et al., 2014a). However, the separation process of HMF from solvent, such as DMSO, is the main drawback due to its high-boiling point, and, hence, alternative solvents (e.g., tetramethylene sulfone) or biphasic solvent systems are actually being explored to simplify the extraction of HMF (Agarwal et al., 2018). Sodium chloride in abiphasic system of water-based solvents has crucial benefits for the selective formation of HMF. An HMF yield of 53% was obtained using a biphasic solvent, comparing AlCl_3_/HCl as the aqueous phase and methyl isobutyl ketone as the organic phase, with the yield reaching 66.2% by adding 20 wt% NaCl (Guo et al., 2020). HMF is, arguably, the most versatile bio-derived platform molecule, but further efforts are needed to increase the yield of low environmental impact strategies.

### Levulinic acid

Levulinic acid (C_5_H_8_O_3_) has been listed as one of the 12 key sugar-derived platform chemicals that can be produced mainly from HMF *via* a rehydration step. It can be used to produce a variety of biochemicals, including biofuels, flavoring agents, plasticizers, polymers, and antifreeze, through different reactions (Liu et al., 2018). Considering the promising potentials of various market applications, the sustainable production of LA from lignocellulosic biomass feedstock *via* biorefinery approaches has attracted significant interest. However, the synthesis of LA from lignocellulosic material subunits usually faces challenges, such as low yield, high equipment costs, severe environmental pollution, and undesired byproducts. The most wildly used pathway for LA production through consecutive one-pot transformation constitutes several steps: (i) the pretreatment of lignocellulosic biomass, (ii) hydrolysis of cellulose to glucose, (iii) isomerization of glucose to fructose, (iv) dehydration of hexoses (glucose and fructose) to HMF, and (v) rehydration of HMF to LA (Deng et al., 2015; Mukherjee et al., 2015). The inevitable formation of humins in these processes can significantly affect the efficiency of biomass. Homogeneous catalysis using SnCl4 allows the efficient production of LA from corncob residue, with a 64.6 mol% yield of LA after 60 min at 180°C (Zhao et al., 2019). However, concerns related to limited catalyst recyclability, reactor corrosion, and waste generation have inspired the development of heterogeneous catalysts (e.g., alkaline-treated zeolites Y) for the produce of LA and its derivatives (Bounoukta et al., 2022). Nevertheless, the production of LA and its derivatives is a promising area that merits further research to economically optimize the whole biorefinery process.

## Current strategies for assessing the feasibility of a lignocellulosic biorefinery

The biofuels produced from various lignocellulosic biomass sources are generally considered clean and eco-friendly energy that can reduce GHG emissions. It is wildly accepted that biofuels and other chemicals derived from biomass residues present a promising route for solving climate change and an energy crisis, and several advanced biofuel companies are working worldwide to seize market share from fossil fuels (Lynd et al., 2017). However, this is not necessarily true due to the tradeoffs among positive and negative effects on the environment (Zucaro et al., 2016). Moreover, many of the biorefinery techniques require further technological innovation, funding, marketing, and lignocellulosic biomass valorization, which still precludes their feasibility on an industrial and commercial scale (Burg et al., 2018; Singhvi and Gokhale, 2019; Bora et al., 2020; Singhania et al., 2022). For this reason, it is necessary to develop a comprehensive assessment framework for the reliable and quantitative evaluation of environmental sustainability.

Life-cycle assessment is a scientifically accepted tool for assessing the possible environmental impacts of a specific product through qualitative, quantitative, confirmable, and manageable information (Singh and Olsen, 2011; Hiloidhari et al., 2017). There are many LCA methods and commercial software available to assess the direct environmental impact and indirect natural resource costs of a defined product or system. Typically, an LCA study consists of four stages. The first stage is the definition of goal and scope, aiming to identify the objective, limits, context, the functional unit, and environmental effects of the system. The second step is life cycle inventory, in which the life cycle of a product is simulated by quantifying all inputs (e.g., materials and energy), outputs (e.g., products and byproducts), and environmental releases (e.g., gas/particle emissions and wastewater). The third step, also known as life-cycle inventory analysis, is a technical process that assesses the potential effects of the inputs and outputs of a defined product. The final step, the interpretation, is an evaluation of all results related to the product, leading to conclusions and recommendations for minimizing the environmental burden. In practice, the type of lignocellulosic raw materials, the applicability of conversion technology, and optimal productivity should be carefully considered to maximize the product yield and profit. Espada et al. (2021) evaluated the environmental feasibility of a cardoon-to-bioethanol process using LCA, suggesting that steam explosion-based processes present the best environmental performance, with a dramatic reduction of primary energy demand (80%) and GHG emissions (45%). Bello et al. (2019) stated that solvents and energy demand are the critical points in the case of producing 2,5-furandicarboxylic acid and HMF from lignocellulosic feedstock. Compared to the conventional thermochemical process, the renewable electricity pathway has lower-energy intensity and a milder environmental impact. Accordingly, Patel et al. (2022) concluded that the processes of HMF production is the most significant factor in overall environmental impact in many categories. A similar study focusing on microalgae biomass, the most promising feedstock for third-generation biofuel production, demonstrated its great potential for bioethanol production, with a favorable net energy ratio (0.45) and net energy balance (–2,749.6 GJ y^–1^), as well as low water and land footprints in bioethanol production plants (Hossain et al., 2019).

A successful biorefinery not only requires promising and eco-friendly process technology but must also include optimized capital investment and operating costs. Techno-economic analysis (TEA) is a crucial approach for the commercialization of bioproducts from lignocellulosic biomass, enabling informed decision-making for process scale-up (Scown et al., 2021). TEA consists of several elements, including process design, process modeling, equipment sizing, a capital cost estimate, and an operating cost estimate, with the aim to recognize the crucial factors, such as the payback period and operating cost. The first TEA of HMF production was performed in 2010, in which the authors claimed that the high price of fructose cannot support a profitable scenario for HMF production from lignocellulosic biomass, no matter how much the other costs are minimized (Davidson et al., 2021). Numerous studies conducted detailed TAE by introducing different strategies, models, and types of biomass material to investigate the economic feasibility and production of biofuels on the industrial scale (Naveenkumar and Baskar, 2020; Aui et al., 2021; Thoppil and Zein, 2021). TEA of bioethanol production from wheat straw revealed that 20 million liters of ethanol could be obtained annually with a sale price equal to or greater than $2.57 L^–1^ (Hasanly et al., 2018). Chong et al. (2020) conducted TEA of third-generation bioethanol production from macroalgae cellulosic residue, finding that 15,833.3 kgh^–1^ of biomass material was required to produce 7,626 kgh^–1^ of bioethanol, leading a net energy ratio of 0.53 and a minimum selling price of $0.54 kg^–1^. The economic analysis of utilization of soybean straw for biofuel production suggested a minimum selling price of $1.94 kg^–1^biohydrogen, with a considerable payback period of 2.5 years (Okolie et al., 2021).

Life-cycle assessment and TEA are both powerful tools to optimize the process configuration and evaluate the potential risks when designing an industrialized biorefinery system. Further investigations are still needed to build a reliable and efficient framework that can ensure the practical application of lignocellulosic biomass and its valorization.

## Conclusion and future perspectives

The low price and broad availability of a wide range of lignocellulosic materials can promote the expansion of the international biofuel market and help reduce carbon dioxide emissions on a global scale.

Nevertheless, an economically and ecologically viable processing methodology has not been fully developed to date. To overcome diverse bottlenecks, further research should consider the following facets: (i) opting for easily converted lignocellulosic materials, (ii) understanding the properties and synergy mechanism of different feedstocks to adopt a proper pre-treatment method, (iii) designing large-scale integration to reduce energy consumption and avoiding unnecessary losses, (iv) optimizing and rationalizing the equipment to enhance process economics, (v) efficient conversion and utilization of hemicellulosic sugars to increase ethanol production; (vi) effective separation and utilization of byproducts and maximization of the recovery of reaction reagents; (vii) promoting vigorous economic and political support to give a competitive edge to the fledgling businesses for the bioconversion of lignocellulose.

## Author contributions

Y-XH conceived the topic. BZ reviewed the literature and prepared the first-draft manuscript. ZC, SY, and Y-XH revised the manuscript. ZC reviewed and edited the content. All authors contributed to the article and approved the submitted version.
